# Predicting Tumor Responses to Gefitinib and Erlotinib

**DOI:** 10.1371/journal.pmed.0020021

**Published:** 2005-01-25

Tyrosine kinases regulate signaling pathways that control cell growth, proliferation, motility, and other critical cellular processes. Mutations in tyrosine kinase genes can lead to abnormal kinase activity, and some tumors become dependent upon this activity for growth and survival. Thus, kinases are attractive targets for anti-cancer drugs. Examples of new kinase inhibitors include gefitinib and erlotinib, which have recently shown promise in treating non-small-cell lung cancer. Unfortunately, gefitinib and erlotinib work only in a subset of patients, and they can have severe side effects, albeit infrequently. So researchers have been trying to find ways to predict who will benefit from therapy with these drugs and who won't.[Fig pmed-0020021-g001]

**Figure pmed-0020021-g001:**
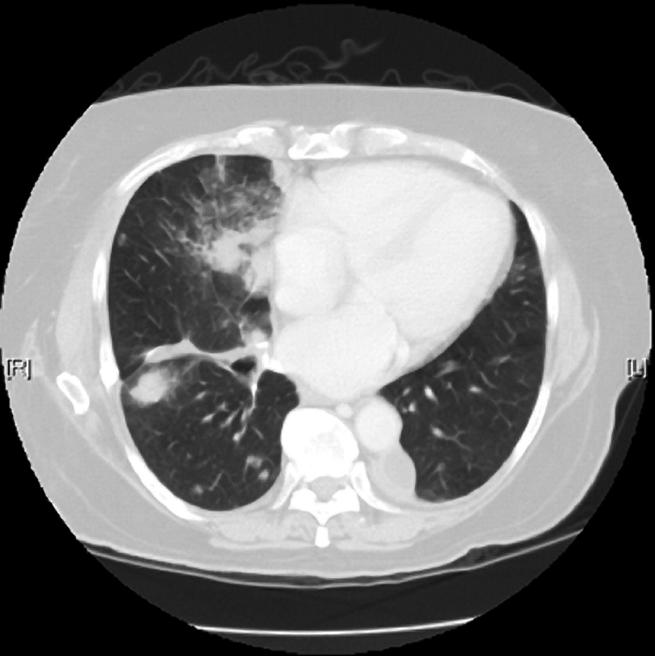
Assessing lung tumors for gene mutations could help guide therapy

Following the work of Lynch et al. (N Engl J Med 350: 2129–2139) and Paez et al. (Science 304: 1497–1500), William Pao and colleagues have previously shown that the epidermal growth factor receptor (EGFR), a tyrosine kinase, is often mutated in non-small-cell lung cancers, and that tumors that harbor such mutations are sensitive to gefitinib and erlotinib.

In this new study, they focused on a signaling protein called KRAS, which functions downstream of many tyrosine kinases, including EGFR. The KRAS gene is also often mutated in lung cancers, but very few cancers have mutations in both EGFR and the KRAS gene. To find out whether KRAS mutations could help to predict which patients would respond to gefitinib or erlotinib, the researchers looked for mutations in EGFR and KRAS genes in 60 tumors for which sensitivity to either drug was known.

They extended their earlier findings that EGFR mutations (which were found in 17 of the tumors) were associated with sensitivity to the kinase inhibitors, and found that tumors that had mutations in KRAS (a total of nine) were refractory (i.e., did not respond) to either drug.

These results need to be validated in larger and prospective trials that use standardized mutation detection techniques. If they are confirmed, knowing the mutation status of EGFR and KRAS in tumors could help physicians decide which patients should receive gefitinib and/or erlotinib. As Inoue and Nukiwa state in a Perspective that accompanies the article, “By combining all the factors that relate to response or resistance, patients who will benefit from treatment can hopefully be identified. Undoubtedly we have taken a great step forward in molecular therapy for lung cancer treatment.”

