# Correction: Predicting Tumor Responses to Gefitinib and Erlotinib

**DOI:** 10.1371/annotation/d3542745-d656-445b-842b-3d338a795bcc

**Published:** 2012-03-05

**Authors:** Predicting Tumor Responses to Gefitinib and Erlotinib

An image that was published alongside another PLoS Medicine article (doi/10.1371/journal.pmed.0020024) and included in the shared PDF download here has been removed from the published PDF because of that image's copyright status. 

